# The Effect of Subglottic Stenosis Severity on Vocal Fold Vibration and Voice Production in Realistic Laryngeal and Airway Geometries Using Fluid–Structure–Acoustics Interaction Simulation

**DOI:** 10.3390/app15031168

**Published:** 2025-01-24

**Authors:** Dariush Bodaghi, Qian Xue, Scott Thomson, Xudong Zheng

**Affiliations:** 1 Department of Mechanical Engineering, University of Maine, Orono, ME 04473, USA;; 2 Department of Mechanical Engineering, Rochester Institute of Technology, Rochester, NY 14623, USA; 3 Department of Mechanical and Civil Engineering, Brigham Young University-Idaho, Rexburg, ID 83460, USA;

**Keywords:** subglottic stenosis, voice production, subject-specific simulation, fluid–structure interaction

## Abstract

This study investigates the impact of subglottic stenosis (SGS) on voice production using a subject-specific laryngeal and airway model. Direct numerical simulations of fluid–structure–acoustic interaction were employed to analyze glottal flow dynamics, vocal fold vibration, and acoustics under realistic conditions. The model accurately captured key physiological parameters, including the glottal flow rate, vocal fold vibration patterns, and the first four formant frequencies. Simulations of varying SGS severity revealed that up to 75% stenosis, vocal function remains largely unaffected. However, at 90% severity, significant changes in glottal flow and acoustics were observed, with vocal fold vibration remaining stable. At 96%, severe reductions in glottal flow and acoustics, along with marked changes in vocal fold dynamics, were detected. Flow resistance, the ratio of glottal to stenosis area, and pressure drop across the vocal folds were identified as critical factors influencing these changes. The use of anatomically realistic airway and vocal fold geometries revealed that while anatomical variations minimally affect voice production at lower stenosis grades, they become critical at severe stenosis levels (>90%), particularly in capturing distinct anterior–posterior opening patterns and focused jet effects that alter glottal dynamics. These findings suggest that while simplified models suffice for analyzing mild to moderate stenosis, patient-specific geometric details are essential for accurate prediction of vocal fold dynamics in severe cases.

## 1. Introduction

Laryngotracheal stenosis (LTS) [1] is characterized by the narrowing of the airway between the larynx and trachea which can occur in the supraglottic, glottic, subglottic, and tracheal regions. It is commonly associated with voice changes or loss, hoarseness, and stridor. LTS can result from prolonged medical intubation, laryngeal trauma, granulomatosis with polyangiitis, or may be idiopathic in nature. The symptoms vary based on the location and severity of the stenosis, but frequently include a breathy, strained voice and increased respiratory effort [2,3].

Narrowing that occurs specifically in the subglottic region is referred to as subglottic stenosis (SGS). In this distinctive disorder, the stenosis is below the true vocal folds and the shape and vibratory properties of the vocal folds themselves remain largely unaffected. Yet, despite anatomically normal vocal folds, breathing difficulty, shortness of breath, hoarseness, and dysphonia are common debilitating symptoms associated with even mild to moderate grades of SGS [4–6]. The precise underlying mechanism of how SGS impacts voice production still remains incompletely understood.

The Myer–Cotton scale is clinically used to classify the severity of SGS based on the narrowing of the subglottic area into four grades: I (0% to 50%), II (51% to 70%), III (71% to 99%), and IV (100% obstruction) [7,8]. While lower-grade SGS (Grade I and II) may require minimal or no treatment, higher-grade SGS (Grade III and IV) often necessitates laryngotracheal resection with reconstruction to restore airway patency and improve voice-related quality of life. Outcomes are generally good with patients experiencing improved breathing and voice. Regular follow-up is important to monitor for potential recurrence [9–12].

Prior studies have attempted to elucidate the interplay between SGS and phonation. Smith and Thomson [13] employed a two-dimensional computational fluid dynamics model to simulate the effects of SGS on vocal fold vibration and voice production. They found that increased airway resistance was the primary factor explaining the minimal effects of SGS up to 60% obstruction, while noticeable changes in voice production occurred at 90% obstruction and beyond. Building on this work, our recent study [14] employed a high-fidelity three-dimensional idealized fluid–structure–acoustic interaction model, confirming that minimal effects persisted up to 75% obstruction, while significant alterations in glottal flow and pressure were evident at 90% and beyond. Additionally, we identified the ratio of the glottis outlet area to the SGS narrowed area as a critical determinant of degree of impact on phonation. Most recently, Hilton et al. [15] validated these computational predictions through an experimental study using a self-oscillating synthetic vocal fold model in conjunction with a subglottic stenosis. Consistent with the simulations, their results demonstrated negligible influence below 80–90% obstruction but significant deviations in aerodynamic, acoustic, and vibratory responses beyond that threshold.

Despite advancements in computational and experimental studies of subglottic stenosis (SGS), several limitations remain. Past studies [13–15] have employed idealized or simplified vocal fold geometries, often relying on two-dimensional models that fail to accurately capture the true three-dimensional nature of fluid–structure interactions. While synthetic vocal fold designs offer valuable insights, they typically lack the precise material properties and layered microstructure of actual human vocal folds. Additionally, neglecting the complex aerodynamic interactions between the glottis, subglottic region, and trachea, as well as the realistic asymmetries of the vocal tract and irregular obstructions, may impact the accuracy of voice production simulations compared to previously studied symmetric idealized airway models [16].

In light of these limitations, there is a pressing need to enhance our understanding of SGS by developing more comprehensive computational models that incorporate accurate vocal fold geometries alongside realistic vocal tract and ambient domains. Our current study addresses this gap by introducing a sophisticated in-house three-dimensional fluid–structure–acoustic interaction model that accurately represents vocal folds, the vocal tract, and the surrounding ambient domain. This improved anatomical precision aims to facilitate a more authentic representation of the intricate fluid and tissue interactions within the laryngeal–tracheal system. By systematically simulating the effects of varying SGS severity on the glottal flow rate, vocal fold vibrations, and acoustic outputs, we aim to elucidate the specific contributing factors and severity thresholds that lead to disrupted voice production associated with SGS.

The paper is structured as follows: Section 2 introduces the governing equations for this in-house model along with details on the vocal fold geometries, material properties, vocal tract, and ambient domain setup. In Section 3, we first present the simulation results without SGS as a baseline, followed by systematic introduction of graded stenosis severities and analysis of the ensuing effects on aerodynamics, vocal fold vibration, and acoustics. Finally, Section 4 summarizes the key conclusions from our study regarding the potential mechanisms underlying the voice changes seen with subglottic stenosis.

## 2. Methods

### 2.1. Governing Equations

This study employs a hydrodynamic/acoustic splitting method to solve the compressible Navier–Stokes equations. Similar approaches have been successfully applied in previous studies [17–22]. This method first decomposes the total flow variables into incompressible and perturbed compressible components as follows:

(1) $$v_{i}=V_{i}+v_{i}^{\prime }\;p=P+p^{\prime }$$

where $v_{i},V_{i}$ and $v_{i}^{\prime }$ represent the total, incompressible, and perturbed compressible velocity components, respectively, while p,P and $p^{\prime }$ represent the total, incompressible and perturbed compressible pressures, respectively. By substituting Equation (1) into the fully compressible Navier–Stokes equations and following the linearization procedure of Seo and Moon [23], the compressible Navier–Stokes equations split into incompressible Navier–Stokes equations and linearized perturbed compressible equations (LPCE):

- Incompressible Navier–Stokes equations:
(2) $$\frac{\partial {\text{V}}_{\text{i}}}{\partial {\text{x}}_{\text{i}}}=0\;\frac{\partial {\text{V}}_{\text{i}}}{\partial \text{t}}+\frac{\partial {\text{V}}_{\text{i}}{\text{V}}_{\text{j}}}{\partial {\text{x}}_{\text{j}}}=-\frac{1}{\rho_{0}}\frac{\partial \text{P}}{\partial {\text{x}}_{\text{i}}}+v_{0}\frac{\partial^{2}{\text{V}}_{\text{i}}}{\partial {\text{x}}_{\text{j}}\partial {\text{x}}_{\text{j}}}$$
- Linearized perturbed compressible equations (LPCE):
(3) $$\frac{\partial v_{i}^{\prime }}{\partial t}+\frac{\partial v_{i}^{\prime }V_{i}}{\partial x_{j}}+\frac{1}{\rho }\frac{\partial p^{\prime }}{\partial x_{j}}=0\;\frac{\partial p^{\prime }}{\partial t}+V_{i}\frac{\partial p^{\prime }}{\partial x_{i}}+\gamma P\frac{\partial v_{i}^{\prime }}{\partial x_{i}}+v_{i}^{\prime }\frac{\partial P}{\partial x_{i}}=-\frac{dP}{dt}$$
  where $\rho_{0}$ represents incompressible flow density, $\nu_{0}$ is the kinematic viscosity, and γ is the ratio of specific heats. By solving these two equations, the incompressible and the perturbed compressible variables are determined, and the total variables are obtained by substituting them back into Equation (1). This hydrodynamic/acoustic splitting method has been successfully verified in simulating acoustics in human phonation against fully compressible flow solutions and theoretical data [24].

The vocal fold dynamics are solved using the Navier equation:

(4) $$\rho \frac{\partial^{2}d_{i}}{\partial t^{2}}=\frac{\partial \sigma_{ij}}{\partial x_{j}}+\rho f_{i}$$

where ρ represents tissue density, d is displacement, σ is stress, and f is the body force per unit mass.

### 2.2. Numerical Algorithms

The incompressible Navier–Stokes equations are spatially discretized using a second-order central difference scheme, employing a cell-centered collocated arrangement for the primitive variables $v_{i}$ and p. Time integration of the incompressible Navier–Stokes equations is performed using the Van Kan fractional step scheme [25]. This scheme consists of three sub-steps:

1. Intermediate velocities are computed by solving a modified momentum equation. The convective terms in this equation are discretized using a second-order Adams–Bashforth scheme, while the diffusion terms are handled with the implicit Crank–Nicholson scheme to alleviate the viscous stability constraint.
2. A pressure correction term is determined by solving the Poisson equation.
3. The pressure and velocity fields are updated based on the derived pressure correction term.

The LPCE is spatially discretized using a sixth-order central compact finite difference scheme [26]. Time integration of the LPCE is accomplished using a four-stage Runge–Kutta method.

An in-house sharp-interface immersed boundary method solver, based on a multi-dimensional ghost cell methodology [27], is employed to handle complex and moving boundaries in the hydrodynamic/acoustic splitting method solvers. In this method, an unstructured triangular surface mesh is immersed within a three-dimensional Cartesian grid, which divides the grid into fluid and solid cells based on their spatial relation to the surface. Solid cells with at least one neighboring fluid cell are designated as ghost cells. A line is drawn from the center of each ghost cell to the surface, perpendicular to it. An image point inside the fluid cells is determined by extending this line until it intersects the surface, with the intersection point positioned midway between the image point and the ghost cell. Flow variables at the image point are specified using bilinear interpolation based on values from adjacent fluid cell nodes. Subsequently, flow variables at the ghost cell are calculated using a central difference approximation along the drawn line to satisfy the boundary conditions at the point of intersection. This procedure ensures second-order accuracy at both solid boundaries and within the computational domain. Further details of this method can be found in Mittal et al. [27].

An in-house finite element analysis code, utilizing a linear elasticity formulation, is employed to solve the Navier equation [28]. The hydrodynamic/acoustic splitting method solvers and the solid solver are explicitly coupled through triangular surface meshes representing a Lagrangian interface. Each time step consists of three steps:

1. Incompressible flow variables are calculated by solving the incompressible Navier–Stokes equations, using the existing deformed shape and velocities of the solid as boundary conditions.
2. Perturbed compressible variables are determined by solving the LPCE, on the same deformed shape. The total forces applied to the vocal fold surface are then computed based on both the incompressible pressure and perturbed pressure from LPCE.
3. The solid solver is executed using the updated total forces, yielding the updated deformed shape and velocities [29,30].

### 2.3. The Subject-Specific Model

**Figure 1.**
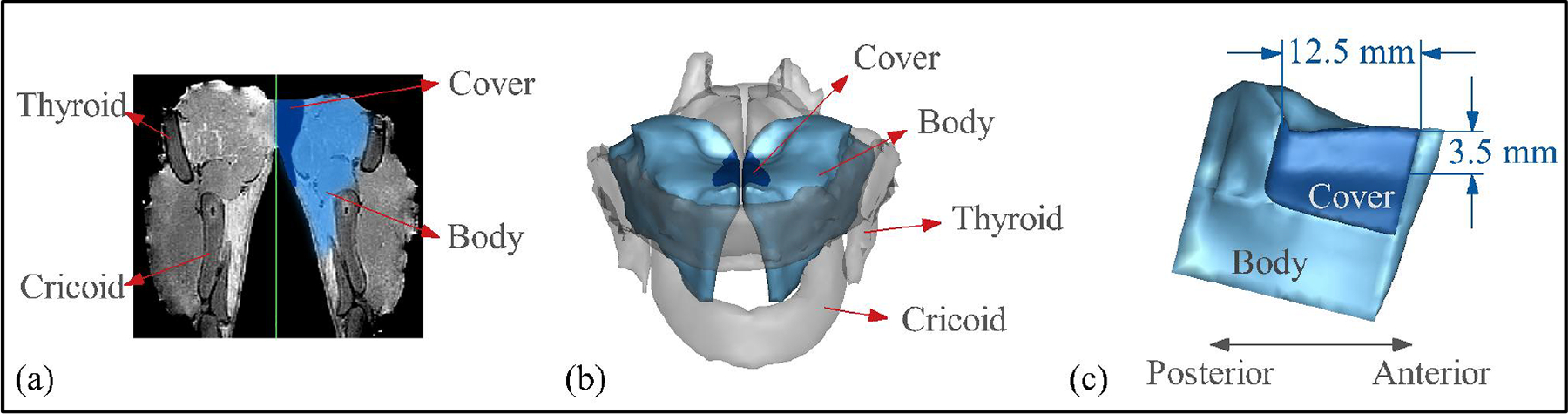
Vocal fold model. (**a**) Vocal fold tissues at a coronal plane of the larynx from the MRI scan [31]; (**b**) the reconstructed three-dimensional vocal folds and cartilages; (**c**) the dimensions of the medial surface of the vocal fold.

A subject specific canine larynx model was adopted from our previous study [31], where the fluid–structure interaction approach was validated against excised laryngeal experiments. This model was reconstructed from a Magnetic Resonance Imaging (MRI) scan of a 20 kg Mongrel canine and consists of a two-layer vocal fold model along with all the major laryngeal cartilages. Figure 1a displays a cross-section of the segmented MRI images for the two-layer vocal fold, while Figure 1b illustrates the reconstructed three-dimensional vocal folds and cartilages. The vocal fold attachments to the cartilages provide the necessary boundary conditions for the solid simulation.

The vocal fold tissues were modeled as transversely isotropic linear viscoelastic materials, with a two-layer inner structure defined using the body-cover labeling scheme, as illustrated in Figure 1c. An inverse method was developed to determine the material properties of the cover and body layers. In this method, a genetic algorithm-based optimization process is employed to determine optimal material properties that match the force-displacement relationship obtained from numerical simulations with the experimental data from Oren et al. [32]. The material properties of each layer used in this study are summarized in Table 1, where E represents the transverse Young’s modulus, E′ is the longitudinal Young’s modulus, G is the longitudinal shear modulus, η is the transverse Poisson ratio, η′ is the longitudinal Poisson ratio, and VSG is the vertical stiffness gradient, which is only defined for the cover layer.

**Figure 2.**
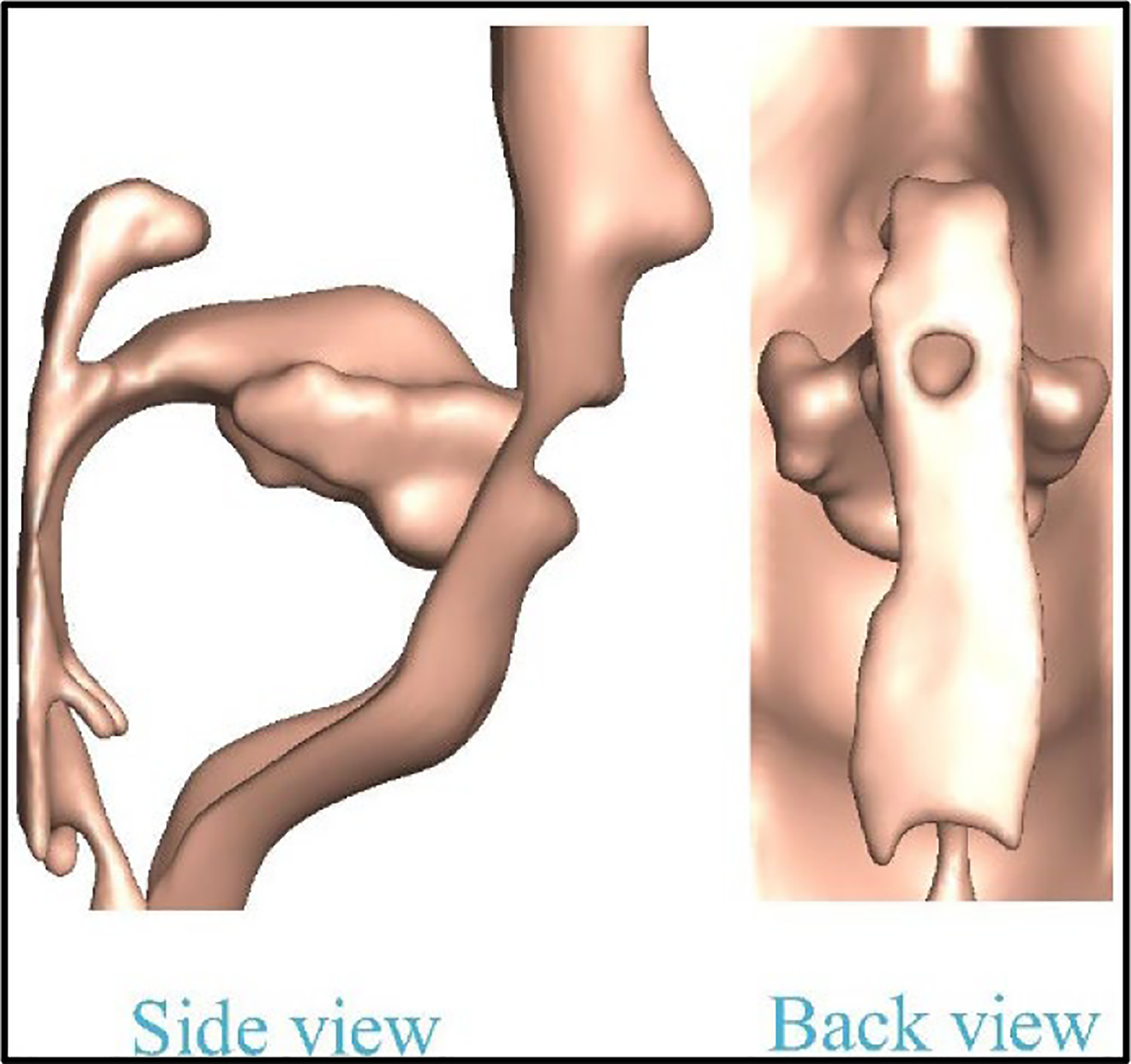
The reconstructed supraglottal tract form a MRI scan of human speaker published by Palo et al. [33].

A realistic supraglottal tract model was adopted from Palo et al. [33], which provides an STL file of a human airway model along with the facial shape of a 30-year-old native male speaker for the Finnish vowel /a/, derived from MRI images. The speaker’s facial shape was also included in the simulation for more realistic boundary conditions for flow and acoustics outside the supraglottal tract. To enhance the model, mesh refinement was performed on the original STL file to generate a smooth surface suitable for flow and acoustic simulations, while also achieving the required surface grid resolution for grid independence. Figure 2 presents side and back views of the reconstructed supraglottal tract and face model. However, it should note that the reconstructed model does not include the shapes of the teeth due to MRI limitations.

**Figure 3.**
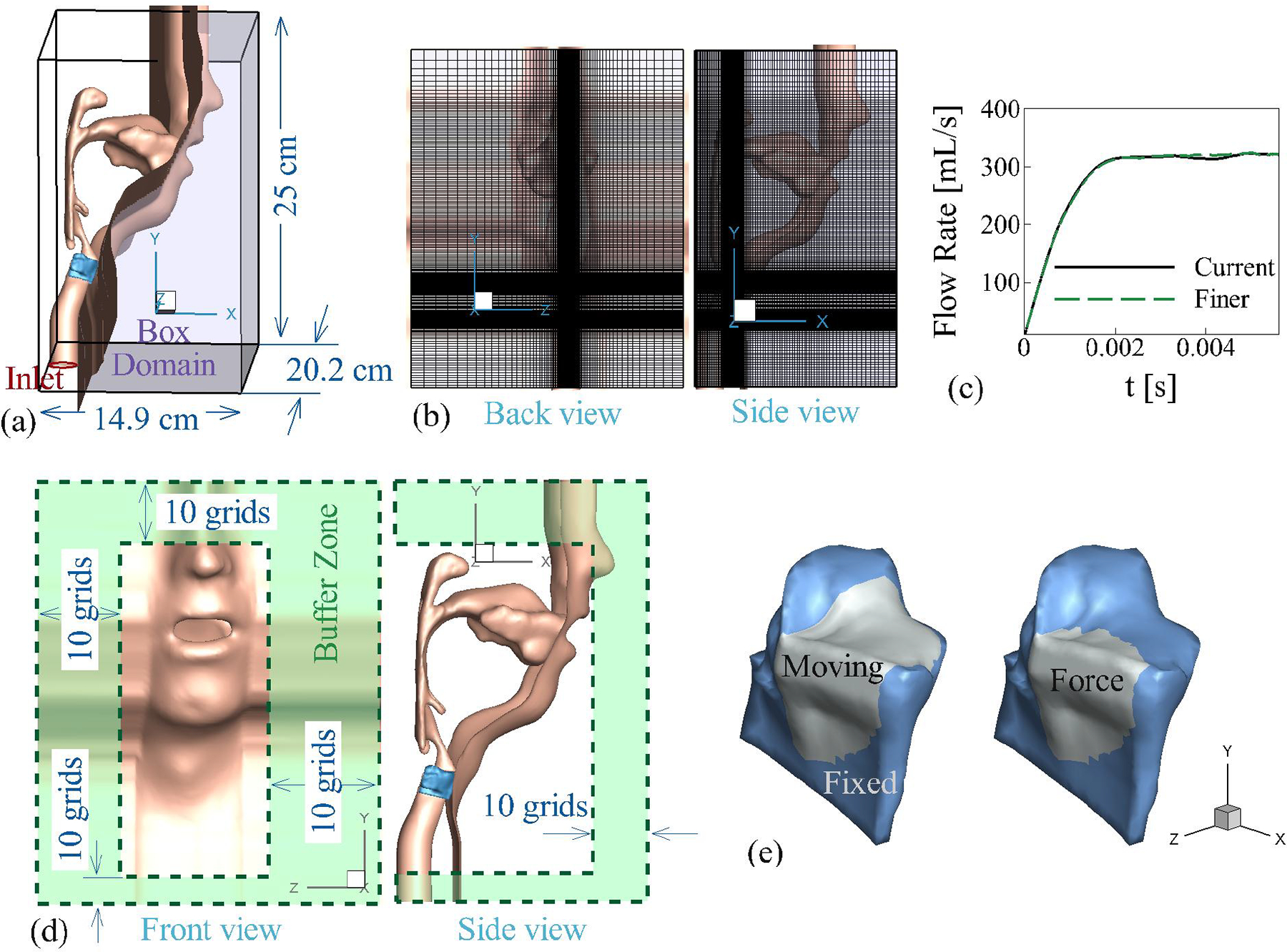
(**a**) The computational domain of the current study; (**b**) domain grid resolution; (**c**) the time history of the flow rate for a stationary vocal fold with a maximum glottal opening for current grids and finer grids measured at the mouth; (**d**) the buffer zones of the LPCE solver; (**e**) boundary conditions of the vocal fold.

In Palo et al. [33], the intraglottal region was not accurately captured due to the rapid vibration of the vocal folds, leading to an approximate representation created by averaging the geometry over the duration of the MRI recording. In the current study, rather than relying on this approximation, we integrate the surface of the reconstructed canine vocal folds into the airway tract model to achieve a more accurate representation of the intraglottal region. The canine vocal fold model was first scaled to match the dimensions of the speaker’s vocal folds and then fused into the speaker model, effectively replacing the approximated vocal folds. Subsequently, the surfaces at the connection points were smoothed to ensure seamless integration. The complete model is illustrated in Figure 3.

### 2.4. Computational Domain and Boundary Conditions

The reconstructed model is immersed in a rectangular domain measuring 14.9 cm × 25 cm × 20.2 cm (Figure 3a), with the walls of the subject’s face extended to the outer domain to ensure solver stability. This configuration creates a sufficiently large environment box that minimizes the influence of outer boundaries on the flow field within the airway tract and prevents reverse flow at these boundaries. Both the incompressible flow solver and the LPCE acoustic solver share the same rectangular domain discretized into a non-uniform Cartesian grid of 128 × 256 × 128 cells, with the highest grid density concentrated around the intraglottal region (Figure 3b). To verify grid independence, a finer grid was employed for the incompressible flow, discretizing the domain into 256 × 256 × 256 cells with a static vocal fold at maximum glottal opening, and as illustrated in Figure 3c, the glottal volume flow rates are nearly identical between these two grid configurations. A Dirichlet pressure boundary condition of 800 Pa is applied at the airway inlet, while a Dirichlet pressure of 0 Pa is set for all outer boundaries of the environment box, as depicted in Figure 3a. To eliminate acoustic reflections from both the airway inlet and the outer boundaries of the environment box, an anechoic buffer zone [34] is enforced at the first and last ten neighboring grid points along these boundaries, as shown in Figure 3d. A non-slip, non-penetration condition is applied to all walls for the incompressible solver, whereas the LPCE solver utilizes a hard wall boundary condition [23]. The density of air is set at 1.1455 kg/m^3^, and the kinematic viscosity is established at 6.6 × 10^−5^ m^2^/s, corresponding to approximately one-fourth of the Reynolds number for normal human phonation, thereby reducing computational costs. This choice primarily impacts the turbulent flow in the supraglottal tract, as the influence of normal pressure on vocal fold vibration significantly outweighs that of viscous pressure, which is less critical in studies of normal phonation.

The vocal folds are discretized into 20,643 tetrahedron elements to accurately capture the complex flow-induced motion during phonation, with a tissue density set at 1.04 × 10^3^ kg/m^3^. Like other state-of-the-art FEM simulations, our model faces inherent challenges in fully representing the physical exchange of structural forces between the vocal folds during contact. To address this limitation, we implement a rigid wall contact model to prevent penetration of the vocal folds, while maintaining a small artificial gap of 0.2 mm between them during closure. While this approach is a numerical approximation of the actual contact mechanics, it enables the successful implementation of the ghost cell methodology and ensures numerical stability. This small gap does result in slight flow leakage and a non-zero glottal area during glottal closure, which are 21% and 20% of the maximum flow rate and glottal area, respectively. Traction boundary conditions are applied at the fluid–structure interaction interface on the free surface, as depicted in Figure 3e. Additionally, zero displacement boundary conditions are enforced on the fixed surfaces, ensuring stable interactions and accurate simulations of vocal fold dynamics.

Small time-steps of 1.149 × 10^−6^ s and 7.181 × 10^−8^ s were employed for the incompressible and LPCE solvers to meet Courant–Friedrichs–Lewy (CFL) stability constraint. The simulation utilized 128 processors on the XSEDE EXPANSE cluster (AMD EPYC 7742 processors), which resulted in a computational time of approximately 16,600 CPU hours for each cycle, which translates to around 129 h of real-time simulation. Simulations have been conducted in a total of 5 cycles.

## 3. Results and Discussion

### 3.1. Baseline Case

In this section, we present the fluid–structure–acoustic interaction (FSAI) simulation results on the original model without introducing subglottic stenosis (SGS). We compared the predicted key phonatory characteristics with the typical physiological range to ensure a reasonable representation of the current model in relation to normal human phonation. The results also serve as baseline values to examine the effects of SGS in the next section.

#### 3.1.1. Baseline Case: Vocal Fold Vibration and Glottal Flow Dynamics

**Figure 4.**
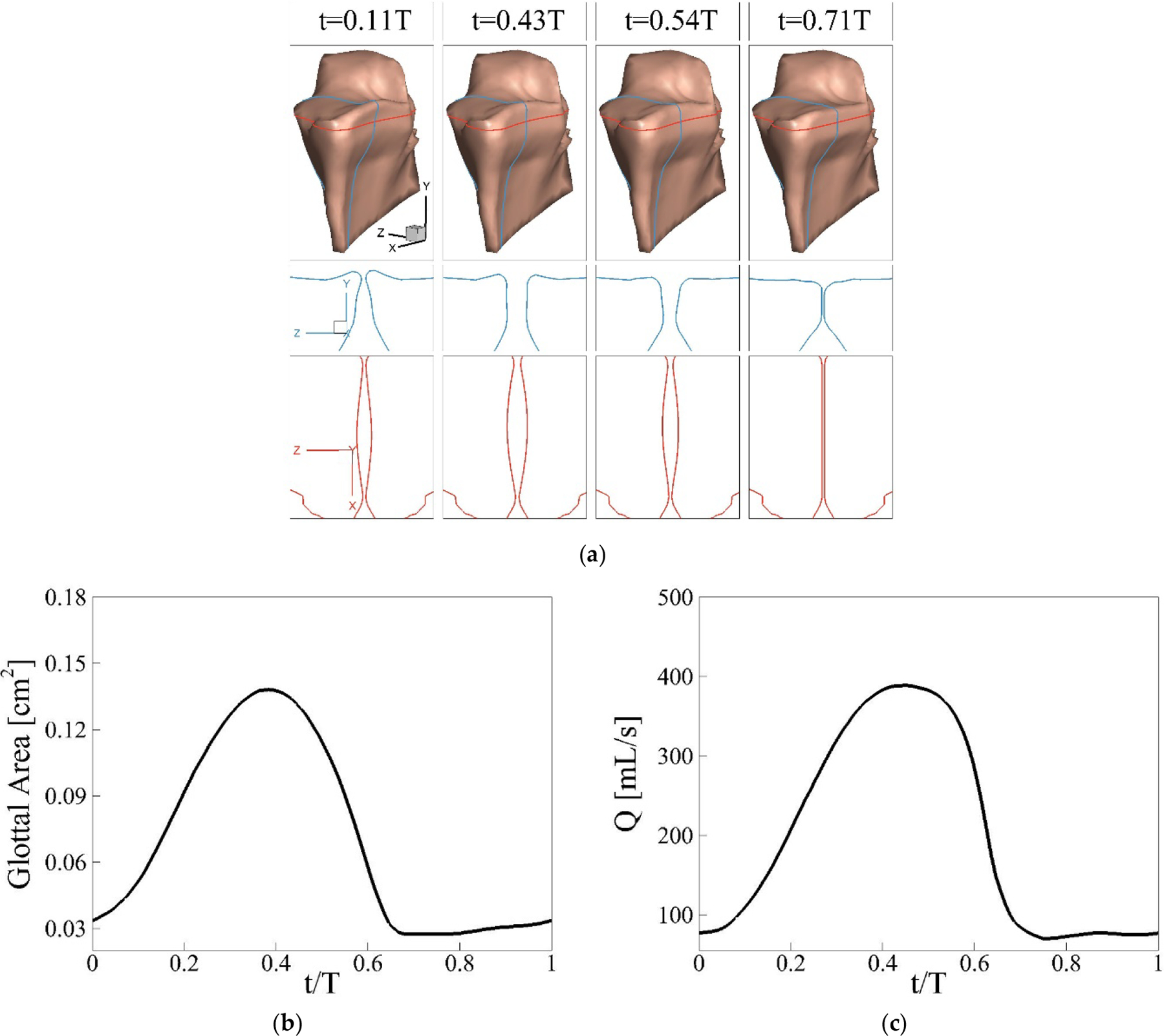
(**a**) The vibration pattern of the vocal fold at different instances during one vibration cycle in the baseline case. The two-dimensional cross-sections of the vocal folds in x-z and y-z planes are also depicted at the same time instances; (**b**) the phase-averaged minimum glottal area waveform and (**c**) the phase-averaged volumetric flow rate waveform over one vibration cycle in the baseline case.

The vibration pattern of the vocal folds at different instances during a single cycle is shown in Figure 4a, along with two-dimensional cross-sections of the folds in the x-z and y-z planes for additional clarity. During typical human phonation, the vocal folds exhibit alternating convergent-divergent glottal shapes, which promote efficient energy transfer from the airflow to sustain vibration [35]. This alternating pattern is clearly observed in the y-z plane, where the airflow initially forces the vocal folds open into a convergent shape (t = 0.11 T), while also pushing the superior surface of the folds upward. At t = 0.4 T, the glottal opening reaches its maximum, forming a straight channel. Following this, the elastic recoil force causes the folds to close, creating a divergent shape (t = 0.54 T), before they eventually reach full closure. A proper orthogonal decomposition (POD) analysis was further conducted to break down the vibrations into their primary eigenmodes, representing the highest energy contributions [36]. The first mode captures the glottal opening-closing motion, accounting for 66% of the total vibration energy. The second mode corresponds to the convergent-divergent motion of the glottis, contributing 26% of the total energy. Together, these two modes encompass 92% of the overall vibration energy, while the remaining 8% is distributed across higher-order modes.

The phase-averaged glottal area and volume flow rate are plotted in Figure 4b,c. The glottal area is defined as the minimum cross-sectional area of the glottal channel, while the glottal volume flow rate is measured at the glottal exit. Phase-averaging is performed by dividing each steady cycle into 1000 intervals, and within each interval, values from multiple cycles are averaged. As shown in Figure 4b, the opening phase occurs from 0 to 0.38 T, reaching a maximum glottal area of 0.138 cm^2^. The closing phase spans from 0.38 T to 0.69 T, with full closure taking place between 0.69 T and T. During closure, a small gap of approximately 0.02 cm^2^ remains, a result of the artificially enforced minimum glottal gap, which is necessary for the successful operation of the ghost cell algorithm in the flow solver. The volume flow rate waveform captures the typical pattern of human phonation, characterized by a slow rise, a rapid decline, and a sustained closure period. The peak and mean values are 388 mL/s and 198 mL/s, respectively. It should be noted that the non-zero flow rate during the glottal closure is due to the enforced artificial minimum gap in the solver. Several key phonatory parameters are further calculated based on the glottal area and volume flow rate. The open quotient $\tau_{o}$, defined as the ratio of the duration of glottal opening to the full cycle duration, is 0.69, which aligns with typical values for normal phonation [35]. The fundamental vibration frequency of the vocal folds is determined to be 154 Hz from a Fourier transform of the glottal area time history. The skewness quotient $\left(\tau_{s}\right)$, defined as the ratio of the glottal flow acceleration duration to the deceleration duration, is found to be 1.32. Additionally, the maximum flow deceleration rate (MFDR), defined as the steepest negative slope of the glottal flow rate, is calculated to be 481 L/s^2^, an important factor in voice intensity [37]. Table 2 compares the typical physiological ranges for normal human phonation parameters with the results of the current simulation. All the simulated values fall well within these physiological ranges [38], indicating that the simulation provides a reasonable representation of normal human phonation.

#### 3.1.2. Baseline Case: Acoustics

**Figure 5.**
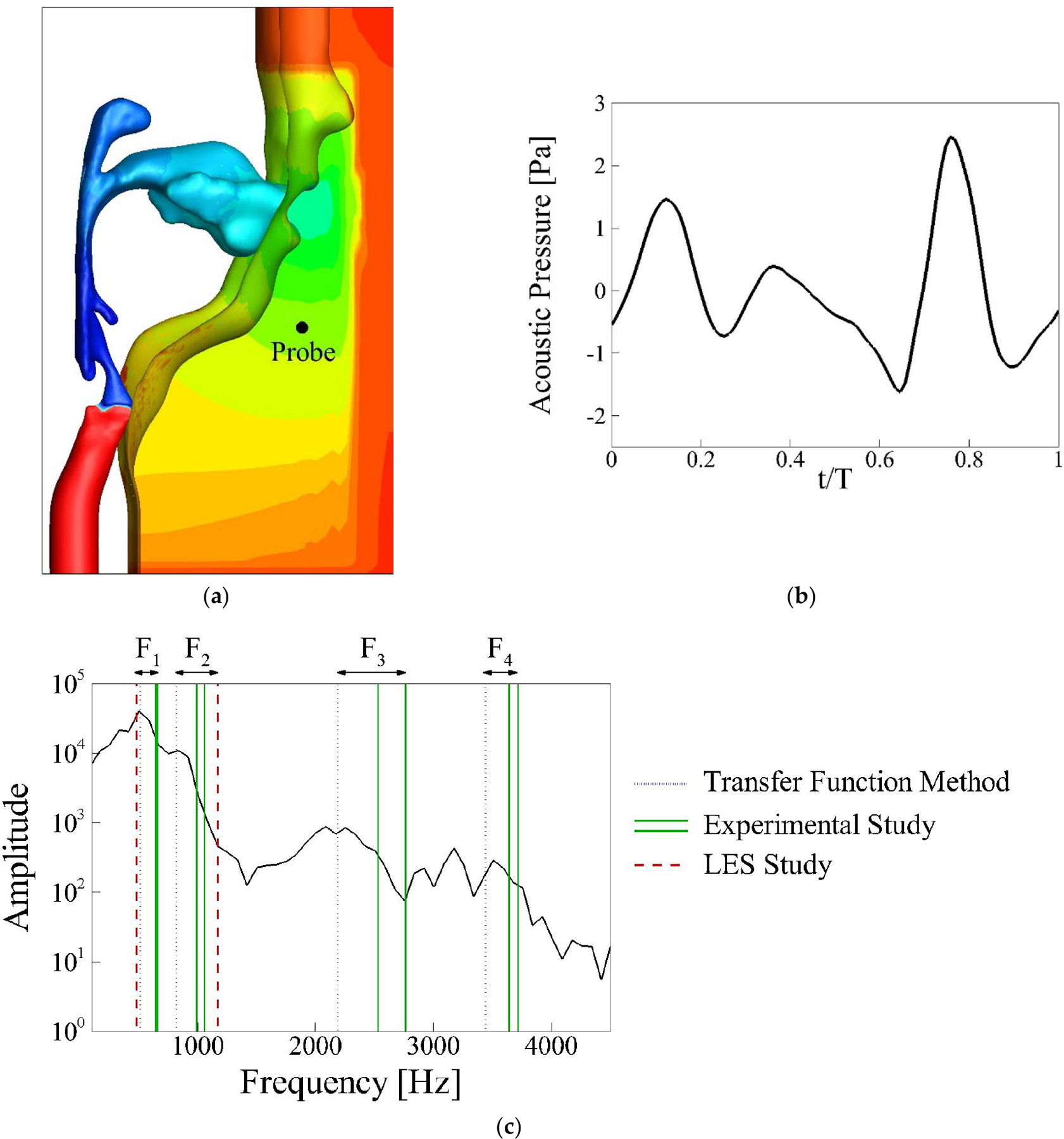
(**a**) Location of the probe 5 cm outside of the mouth; (**b**) the phase-averaged acoustic pressure waveform at this probe over one vibration cycle in the baseline case; (**c**) the pressure Fourier transform spectra, highlighted with calculated formants using the transfer function method, experimental study [33], and LES study [43].

Figure 5a shows the acoustic pressure contour at a specific time instant, illustrating the overall distribution of acoustic pressure. A probe placed 5 cm from the mouth, as indicated in Figure 5a, was used to record the acoustic signals. The phase-averaged acoustic pressure recorded at this location is shown in Figure 5b. Focusing on t/T = 0.7, the pressure rises sharply, reaching a peak with a maximum value of approximately 2 Pa. This peak corresponds to the moment when the vocal folds are closing and the volumetric flow rate deceleration is maximum, which occurs around t/T = 0.6. The phase difference of around 0.1 is due to the time required by the sound to travel from glottis to the probe. Following this, a second peak occurs around t/T = 0.15, though lower in magnitude than the first. The amplitude of the oscillations continues diminishing after the second peak, reflecting the damping effect in the supraglottal tract. Overall, the curve captures the typical oscillatory pattern of acoustic pressure during phonation, with noticeable damping as the energy of the sound waves dissipates towards in the cycle [39–41]. In our previous study utilizing simplified geometries [24], the acoustic pressure also exhibited approximately three oscillations per vibration cycle. However, in the current study, the amplitude of the acoustic pressure peaks decreases more significantly within the same cycle. This discrepancy in acoustic pressure amplitude can be attributed to the differing boundary conditions used in the two studies. In the previous work, a total reflection boundary condition was applied at the mouth, causing all incident waves to be reflected, resulting in higher amplitudes. In contrast, the present study incorporates the full mouth shape, along with a box domain outside the mouth, allowing for partial dissipation of the incident waves at the mouth opening. This partial dissipation leads to lower amplitudes in the second and third peaks of the acoustic pressure wave observed in the current simulation.

To analyze the acoustic characteristics of the vocal tract, we calculated the formant frequencies using a method based on the source-filter mechanism [42], as introduced by Seo and Mittal [26]. This method defines a transfer function (TF) that represents the formant frequencies:

(5) $$\text{TF}(f)=\frac{\Delta p(f)}{\left[\partial Q/\partial t](f\right)}$$

where these variables are in the frequency domain. Figure 5c shows the formant frequencies obtained using the TF method, alongside the corresponding values for the same supraglottal tract reported in the experiment study by Palo et al. [33] and LES study by Schickhofer et al. [43] for comparison. The Fourier transform spectrum of the acoustic pressure from our simulation is also overlaid to facilitate discussion. The first four formants from the TF method are F1 = 510 Hz, F2 = 818 Hz, F3 = 2186 Hz, and F4 = 3441 Hz. These formants correspond to distinct peaks in the Fourier transfer spectrum. The LES study by Schickhofer et al. [43] reported F1 = 480 Hz and F2 = 1170 Hz, while the experiment study by Palo et al. [33] reported ranges of F1 = 644–658 Hz, F2 = 989–1059 Hz, F3 = 2530–2763 Hz and F4 = 3643–3715 Hz. Our predictions using the TF method are reasonably close to these values. In addition to formant frequencies, the spacing between formants is also important to quantify vocal tract acoustics. In our study, the distances between F1–F2, F2–F3, and F3–F4 are 308 Hz, 1368 Hz, and 1255 Hz, respectively. These values align closely with the experimental ranges of 331–415 Hz, 1471–1774 Hz, and 880–1185 Hz. The F1–F2 distance is notably smaller than the 690 Hz reported in the LES study. In summary, the formants prediction of the TF method, including both formant frequencies and spacing between them, show good agreement with the experimental study.

To further characterize the acoustic output of our model and validate its physiological relevance, we calculated additional acoustic parameters including the sound pressure level and vocal efficiency. The sound pressure level is calculated using:

(6) $$SPL=20{log}_{10}\left(\frac{RMS(p)}{p_{0}}\right)$$

where $p_{0}=2\times 10^{-5}$ Pa [35]. Based on the pressure-distance relationship, where acoustic pressure $p\propto r^{-1}$ [14], we estimated the sound pressure level at a distance of 30 cm from the mouth to be approximately 73.78 dB, which aligns with typical human phonation ranges [35,44,45].

The vocal efficiency is calculated by:

(7) $$\eta_{v}=\frac{4\pi r^{2}10^{(SPL-120)/10}}{P_{pul}U_{g}}$$

where $P_{pul}$ is the pulmonary pressure, $U_{g}$ is the mean glottal flow rate [35]. This results in a vocal efficiency of 6.47 × 10^−4^% for the current case, which is in a typical range of human phonation [35].

### 3.2. SGS Cases

#### 3.2.1. SGS Cases: Model

**Figure 6.**
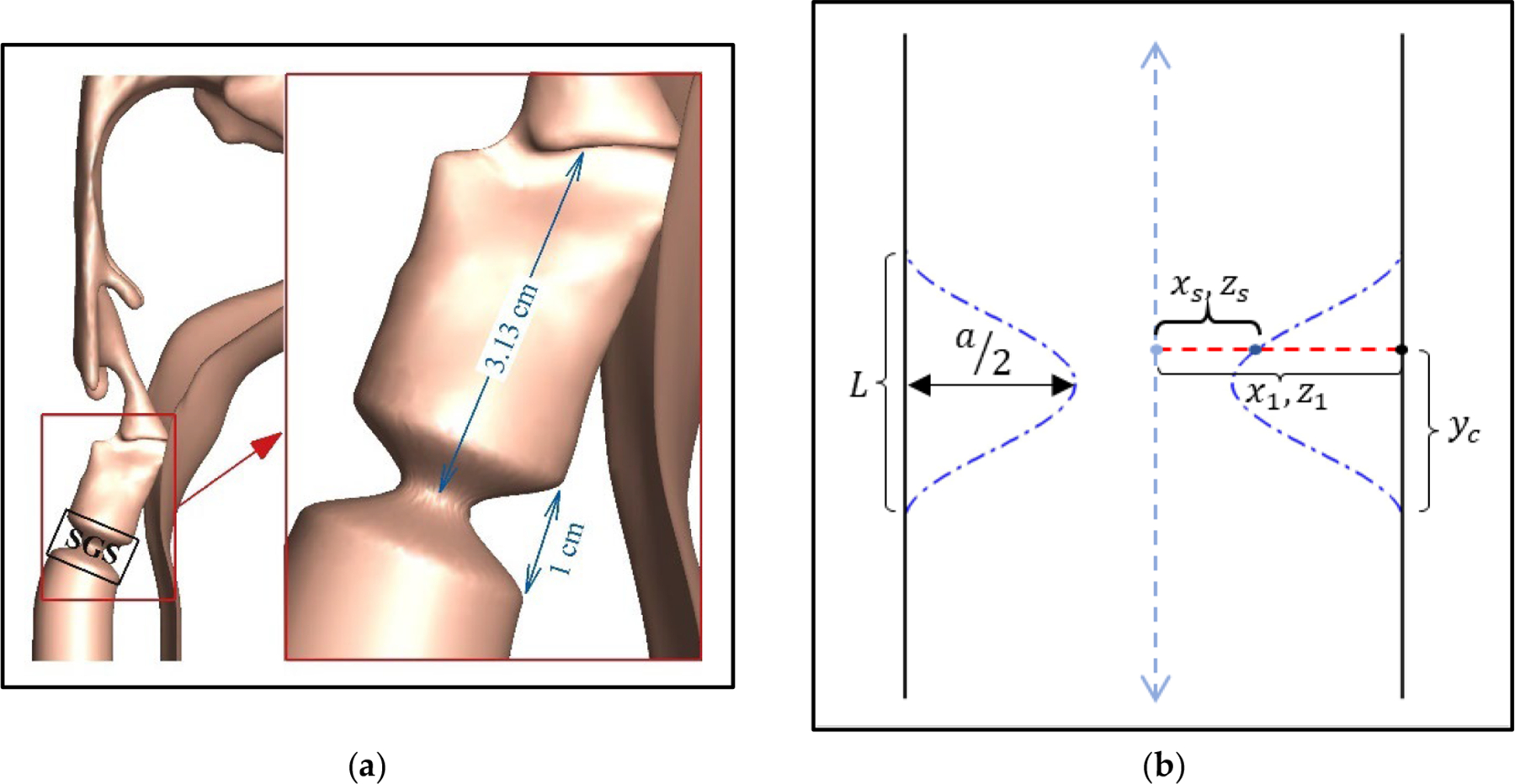
(**a**) The SGS location and the axial distance between the narrowest section of the SGS and superior surface of the vocal folds; (**b**) schematics of the cross-section of an idealized SGS geometry and the parameters used in the Equation (8) to create the SGS.

Building on our previous study [14], an idealized cosine function is employed to simulate the subglottic stenosis (SGS) in the subglottal tract (Figure 6a). To generate the SGS, we first rotate the entire tract counterclockwise by 22° around the z-axis, aligning the subglottal tract with the y-axis, as shown in Figure 6b. We then apply Equation (8) to all tract nodes within the SGS range:

(8) $$x_{s}=x_{1}-x_{1}\times \frac{a}{2}\times \left(1-\text{cos}\left(2\pi \frac{y_{c}}{L}\right)\right)\;z_{s}=z_{1}-z_{1}\times \frac{a}{2}\times \left(1-\text{cos}\left(2\pi \frac{y_{c}}{L}\right)\right)\;y_{s}=y_{1}$$

where $\left(x_{1},y_{1},z_{1}\right)$ are the original coordinates of the undeformed tract node. $y_{c}$ is the distance between the lower bound of the SGS and $y_{1}.\;L$ is the SGS length, and a is the percentage reduction of the hydraulic diameter of the tract cross-section due to the SGS. $\left(x_{s},y_{s},z_{s}\right)$ are the new coordinates of the node after applying SGS (Figure 6b). Lastly, we rotate the whole tract back clockwise by 22° to its original orientation. The resulting shape is shown in Figure 6a.

As shown in our previous study [14], vertical location of the SGS yields negligible effects on phonation. Therefore, the SGS location in current study, defined as the distance between the superior surface of the vocal folds and the narrowest section of the SGS is kept at 3.13 cm, which is the average value observed in 92 SGS patients [46,47]. Additionally, the most common SGS length was reported to be 1.0 cm [46], and this value is used as the length L in our model. To study the effect of SGS severity, we simulated five SGS severities: 0% (baseline case), 50%, 75%, 90%, and 96%, where the severity level corresponds to the percentage of area reduction due to SGS. These area reductions are applied by adjusting the value of a (as defined in Equation (8)) to 0, 0.2929, 0.5, 0.6838, and 0.8, respectively.

#### 3.2.2. SGS Cases: Vocal Fold Vibration and Glottal Flow Dynamics

**Figure 7.**
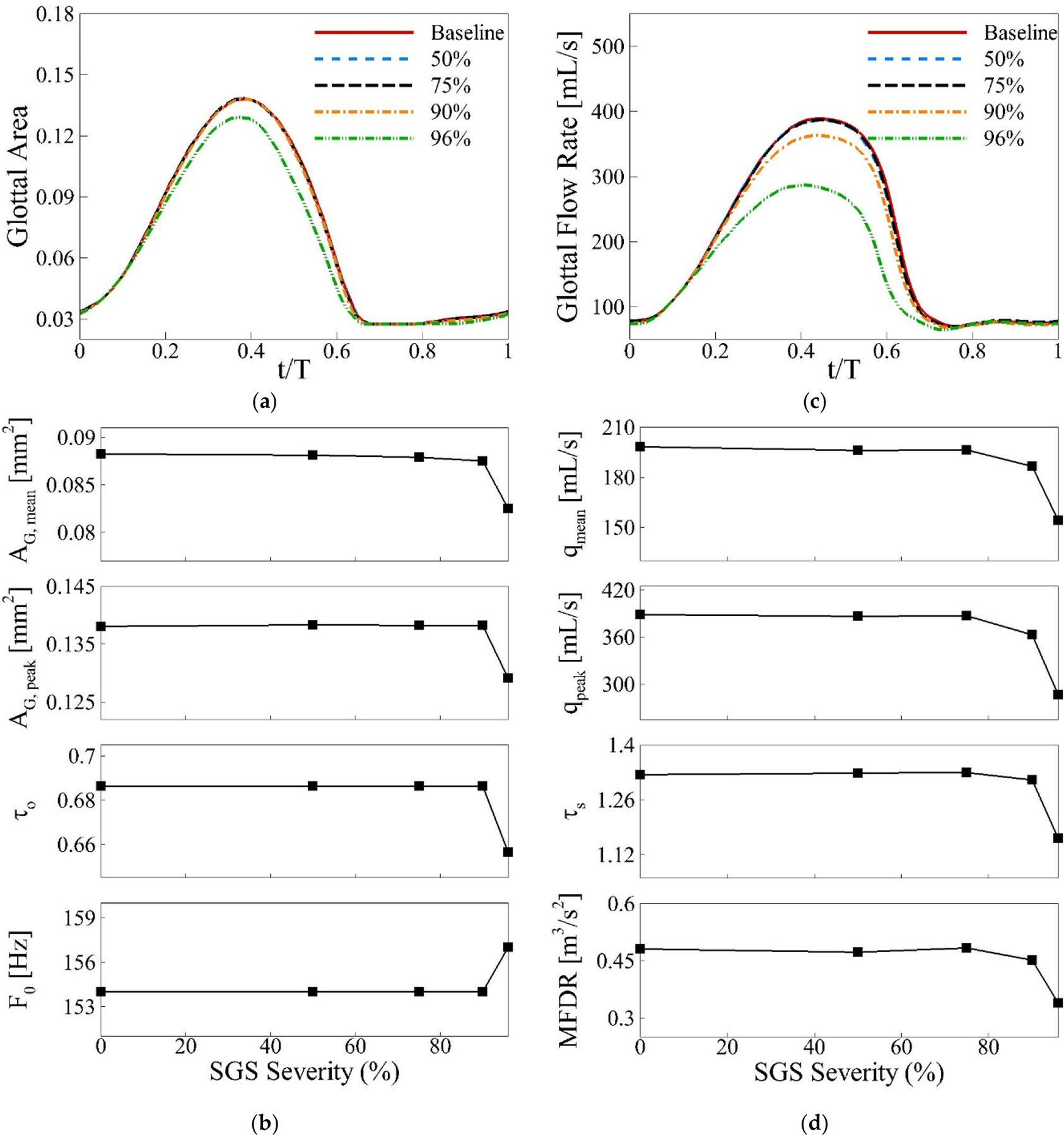
(**a**) The phase-averaged glottal area waveform, (**b**) the mean glottal area, peak glottal area, open quotient, and fundamental frequency; (**c**) the phase-averaged flow rate waveform; and (**d**) the mean flow rate, peak flow rate, skewness quotient, and maximum flow deceleration rate (MFDR) for different SGS severity cases.

The phase-averaged glottal area waveform for all cases is shown in Figure 7a. The waveform remains unchanged for severities below 90%, indicating that the threshold for significant changes in the glottal area occurs at 90% severity. For the 96% severity case, there is a noticeable decrease in both the mean and peak glottal areas, along with a slight leftward shift in the peak glottal area. The open quotient $\tau_{o}$ and fundamental frequency $f_{0}$ both decrease as the severity increases beyond 90%, with the most significant changes occurring at the 96% severity level (Figure 7b). The phase-averaged volumetric flow rate at the glottis is shown in Figure 7c. Changes in the flow rate remain negligible for SGS severities up to 75%. However, beyond 75% severity, the flow rate begins to drop significantly. The mean and peak flow rates decrease rapidly as SGS severity increases, with a 22% reduction in the mean flow rate and a 26% reduction in the peak flow rate for the 96% severity case. Additionally, there is a slight leftward shift in both the peak flow rate (by 0.04T) and the closure instance (by 0.02T) for severe SGS cases. This leads to a shorter flow acceleration duration and a longer flow deceleration duration. The decreases in peak flow rate and the increased closure duration for high SGS severities suggest a drop in the maximum flow declination rate (MFDR). Figure 7b confirms this, showing a maximum MFDR reduction of 30% for the most severe case. Significant changes in the flow rate begin at 75% SGS severity, which corresponds to Grade III and Grade IV SGS categories. This threshold differs from the changes observed in vocal fold dynamics, where significant alterations do not occur until 90% severity. This difference is consistent with our earlier findings, likely resulting from the distinct physical mechanisms governing these phenomena. The flow rate is more sensitive to stenosis due to increased viscous losses as air passes through the narrowing airway, making flow characteristics change at lower severities. In contrast, vocal fold vibration depends primarily on pressure distribution around the folds, which remains stable enough to maintain consistent vibration patterns until more severe stenosis occurs (90% and above).

In comparison with our previous study using simplified geometry [14], several differences emerge, primarily due to the use of patient-specific airway geometry and the inclusion of a realistic environmental flow domain with an anatomically accurate facial structure. In the previous study, the peak glottal area for the 96% severity case exhibited a slight rightward shift, whereas in this study, it shifts to the left. Likewise, the open quotient $\tau_{o}$ increased for 96% and 99% SGS severities in the previous study, but in the current work, it decreases for the 96% severity case. Additionally, the fundamental frequency $f_{0}$ decreased for both 96% and 99% severities in the earlier study, whereas it increases for the 96% severity case in the present study. Regarding glottal flow, the previous study demonstrated a constant flow acceleration duration across all SGS severities, with only a slight increase in deceleration duration. In contrast, the current study reveals a reduction in flow acceleration duration and a more pronounced decrease in deceleration duration for severe SGS cases. These combined effects lead to a larger drop in the speed quotient $\tau_{s}$ for the 96% severity case in this study (0.162) compared to the previous study (0.035).

**Figure 8.**
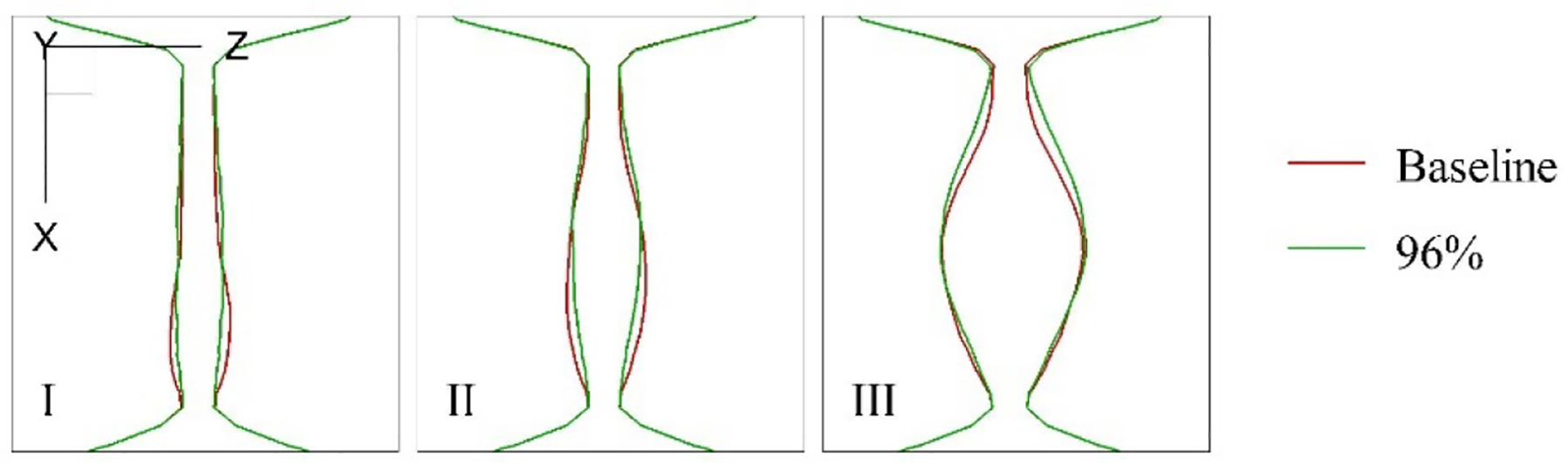
The x-z plane cross-section of the vocal fold vibration at y = 7.6 cm during the beginning of the opening for the baseline and 96% severity cases. The z-axis is magnified five times to visualize the difference between the two cases.

To better understand the differences between the current study and our previous work regarding peak glottal area timing, $\tau_{o}$, and $f_{0}$, we examine the x-z plane cross-section of vocal fold vibration at y = 7.6 cm during the initial opening phase. Figure 8 illustrates this comparison for both the baseline and 96% severity cases, with the z-axis magnified fivefold to emphasize the differences. In the baseline case, the opening process begins at the anterior section of the vocal fold (Step I), followed by a progressive movement towards the middle of the vocal folds in Steps II and III. This pattern is consistent with the findings of Xue et al. [16] in their study of subject-specific laryngeal dynamics. The earlier opening at the anterior end, compared to the posterior end, can be attributed to the material incompressibility, which causes a delay in the overall opening process. In contrast, for the 96% severity case, the opening occurs more uniformly across the middle of the vocal fold throughout all steps. We hypothesize that this difference is due to the more focused jet resulting from the subglottic stenosis (SGS) narrowing, which applies higher pressure to the central portion of the vocal fold. This concentrated pressure causes a slightly faster opening for the 96% severity case, leading to a leftward shift in the timing of the peak glottal opening. Consequently, we observe slight decreases in $\tau_{o}$ and corresponding increases in $f_{0}$. This shift in dynamics highlights the impact of stenosis-induced airflow patterns on vocal fold vibration and the resultant phonatory characteristics.

This shows that the above discrepancies can be attributed to the use of patient-specific airway geometry and vocal folds. The more complex model captures subtle interactions between the airflow and the vocal fold surfaces that were not present in the simplified model [14]. Furthermore, the realistic geometry leads to different pressure distributions and flow patterns within the larynx, particularly in severe SGS cases. The focused jet in the patient-specific model creates a more concentrated pressure on the vocal folds, altering their vibratory patterns. This change in dynamics highlights the impact of stenosis-induced airflow patterns on vocal fold vibration and the resultant phonatory characteristics, emphasizing the importance of using detailed, patient-specific models for accurate predictions in cases of severe SGS.

**Figure 9.**
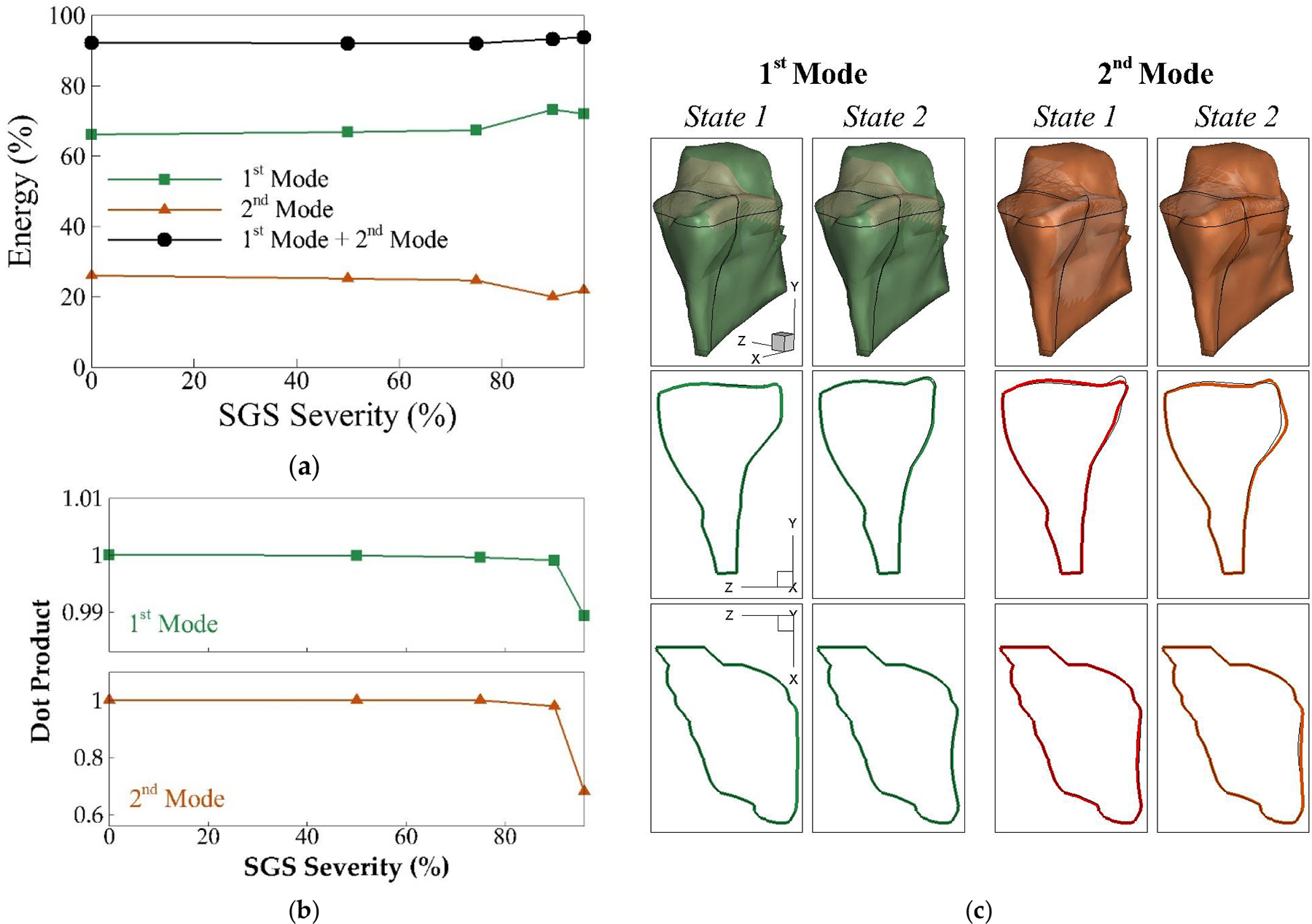
(**a**) The energy percentage of the two dominant proper orthogonal decomposition (POD) modes of the vocal fold vibration versus SGS severity in the parametric cases; (**b**) the similarity of the corresponding POD modes of the vocal fold vibration versus SGS severity in different parametric cases; (**c**) the three-dimensional shape of these POD modes for the baseline case and the 96% SGS severity case; 2D cross sections of each mode shows the two extreme phases of the mode; narrow lines are baseline case and thick line is 96% case.

Figure 9 presents the results of the 1st and 2nd modes from the POD analysis for our current study, which together account for approximately 90% of the total energy. Figure 9a illustrates the energy distribution of each mode and the sum of the first two modes’ energies across different SGS severities. For severities below 75%, the energy distribution remains relatively constant. However, in the 90% and 96% severity cases, significant changes are observed: the energy of the 1st mode increases by 11% and 9%, respectively, while the energy of the 2nd mode decreases by 23% and 16%. Despite these notable shifts, the combined total energy of the first two modes sees only a slight increase, remaining under 2%.

To evaluate the consistency of mode shapes across different SGS severities, the dot product of each mode shape with its corresponding baseline case mode shape was calculated, as shown in Figure 9b. A dot product value of 1 indicates identical modes, while 0 indicates orthogonal modes. Our analysis reveals that the 1st mode shape remains largely unchanged across all SGS severities. However, the 2nd mode shape undergoes significant alterations in the 96% severity case, where the dot product drops to 0.68, suggesting a substantial deviation from the baseline mode shape.

Figure 9c illustrates the two extreme states (referring to two peak displacement state during a vibration cycle generated by each POD mode) of the 1st and 2nd shapes for both the baseline and 96% severity cases, along with two-dimensional cross-sections of the vocal fold in the y-z and x-z planes. Comparing these two severity cases, we observe that the 1st mode remains relatively stable, while the 2nd mode shape exhibits noticeable differences between the baseline and 96% severity cases.

It’s important to note that while the 90% severity case maintains mode shapes similar to the baseline, we observe an increase in the 1st mode’s energy and a decrease in the 2nd mode’s energy (Figure 9a). This suggests that for the 90% case, the lateral motion strengthens while the convergent-divergent motion weakens. For the 96% case, although the energy distribution is comparable to the 90% case, we see more substantial changes in the mode shapes, particularly in the convergent-divergent motion. These findings align with our previous study, confirming that vocal fold vibration is significantly affected by SGS severities above 90%. These higher severities correspond to Grade III and Grade IV SGS classifications, underscoring the clinical relevance of these observations.

#### 3.2.3. SGS Cases: Acoustics

**Figure 10.**
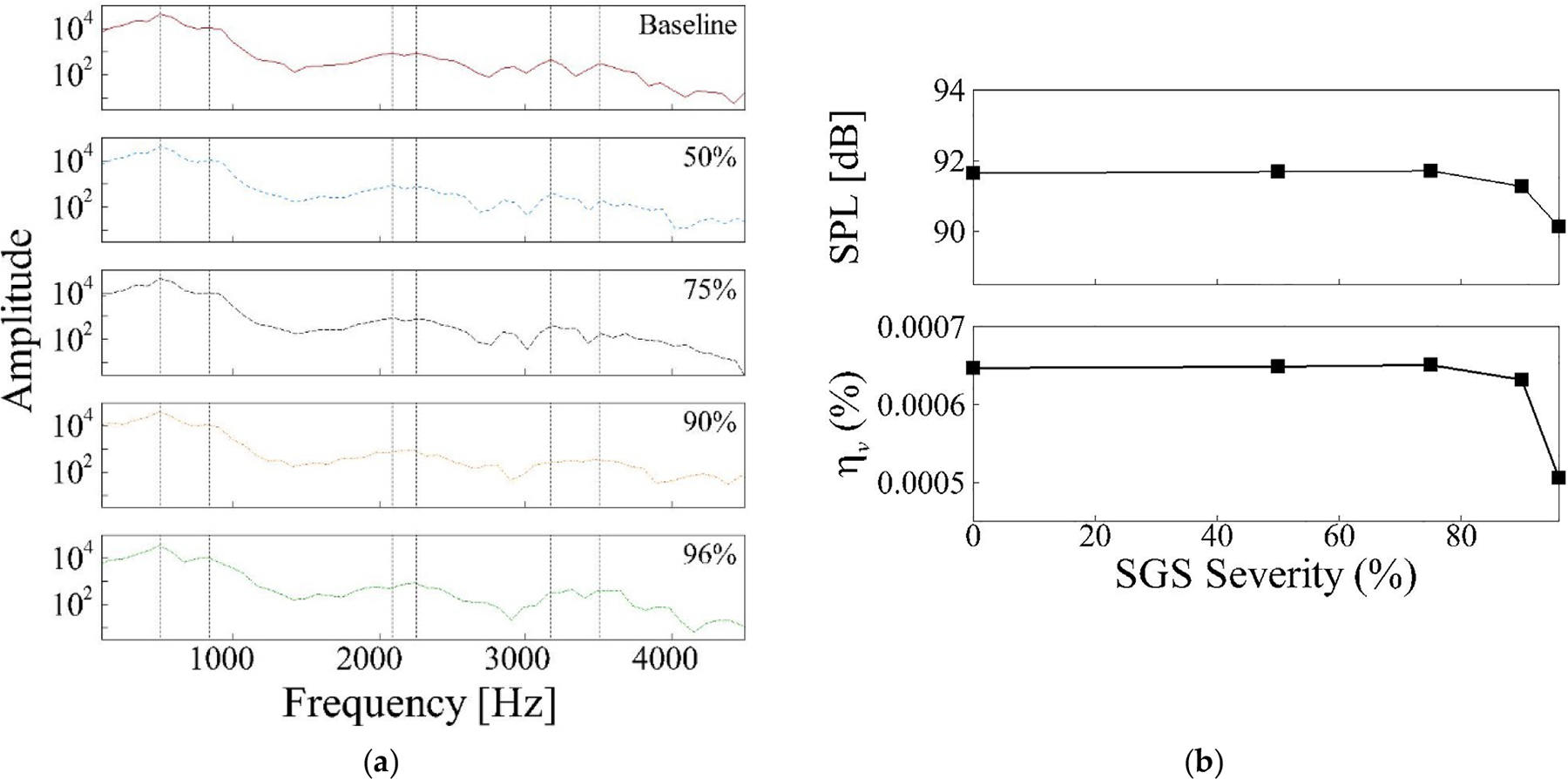
(**a**) The Fourier transform of the acoustic pressure at the probe 5 cm outside of the mouth in the parametric cases; (**b**) the sound pressure level and vocal efficiency for different SGS severities.

The effect of SGS severity on voice is first quantified by calculating the Fourier transform of the acoustic pressure, using the same procedure introduced for the baseline case at the probe 5 cm outside of the mouth, plotted in Figure 10a. This plot shows that the first two peaks remain consistent in frequency for all cases. For the 50% and 75% severities, the behavior of the spectrum and the peaks is similar to the baseline case. However, for 90% and 96% severities, the third and fifth peaks observed in the baseline case are significantly reduced or absent, and the peaks are redistributed. The suppression of these spectral components in severe SGS cases can be attributed to the increased viscous losses in the narrowed region. These acoustic changes align with our earlier POD analysis results. For the 90% and 96% SGS severities, we observed changes in the energy distribution of the first and second POD modes, indicating a shift in the dominant vibration patterns. Furthermore, for the 96% severity case, we noted slight changes in the mode shapes themselves. These findings suggest the idea that severe stenosis alters the fundamental vibration characteristics of the vocal tract, which in turn affects the acoustic output. These combined observations suggest that, similar to the glottal flow results, the SGS severity effect on acoustics is not noticeable up to 75% severity and becomes significant beyond that threshold. To further investigate this, we calculated the sound pressure level and vocal efficiency for different cases (Figure 10b). The results show that for higher SGS severities, sound pressure level decreases by 0.4 and 1.5 dB for 90% and 96% severity cases, respectively. Moreover, using $\frac{\eta_{v,\text{baseline}}-\eta_{v,\text{SGS}}}{\eta_{v,\text{baseline}}}\times 100$ we found a decrease in vocal efficiency of 2.3% and 21.8% for 90% and 96% SGS severities, respectively.

These results agree with our previous study’s findings [14], where similar changes in these parameters were observed for SGS severities of 90% and above. The more pronounced reduction in vocal efficiency, compared to sound pressure level, for severe SGS cases is likely due to the combined effects of altered aerodynamics and the acoustic properties of the vocal tract.

#### 3.2.4. SGS Cases: Underlying Mechanism

**Figure 11.**
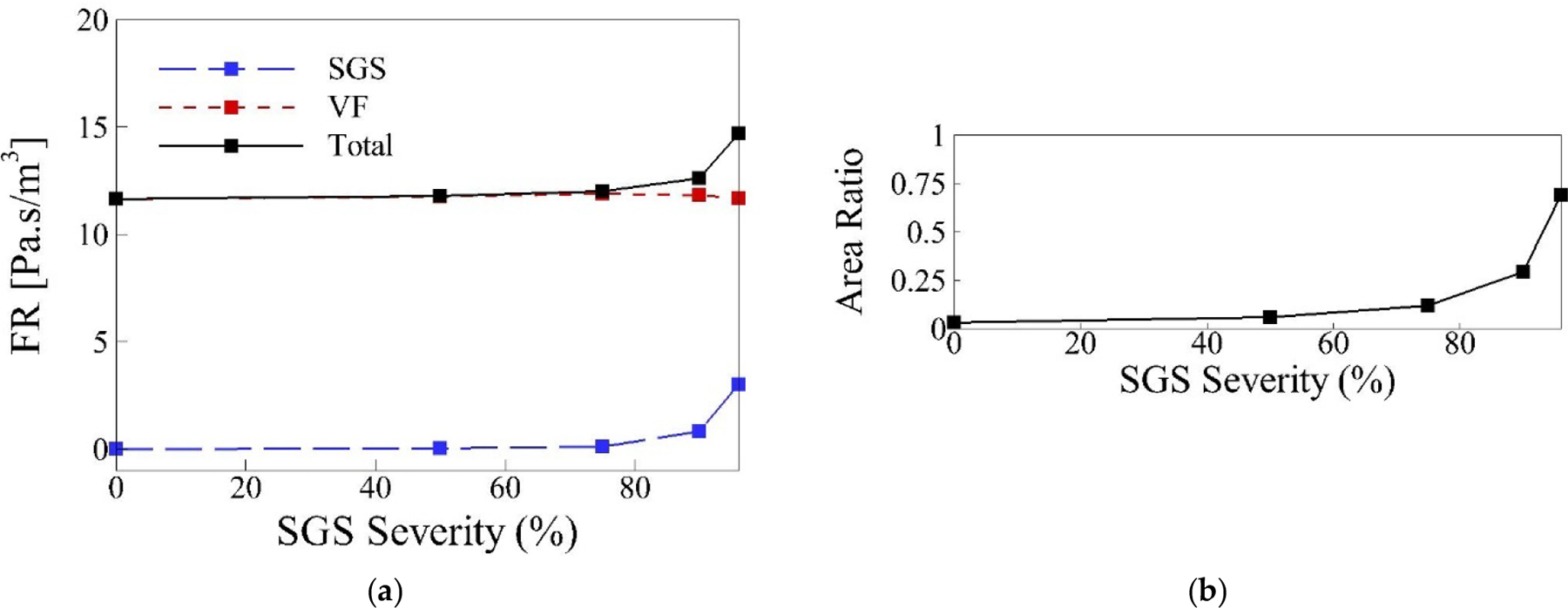
(**a**) The cycle-averaged flow resistance across the SGS, vocal folds and the summation of these two versus SGS severity in the parametric cases; (**b**) the ratio of the cycle-averaged glottal area to the SGS area versus SGS severity in the parametric cases.

We examine the correlation between flow resistance, area ratio, and SGS severity effects on voice production. We define flow resistance as $\frac{\left|\Delta p_{i}\right|}{Q}$, where $\Delta p_{i}$ is the pressure difference across the SGS, glottis, or their summation (represented by i), and Q is the corresponding flow rate. Figure 11a plots the cycle-averaged flow resistance across the SGS ($FR_{SGS}$), across the glottis ($FR_{VF}$) and their sum ($FR_{\text{Total}}$) for different SGS severity cases. Figure 11b shows the area ratio, defined as the ratio of the glottal area to the SGS area, plotted against SGS severity.

For SGS severities below 75%, $FR_{SGS}$ is negligible, making $FR_{\text{Total}}$ essentially equal to $FR_{VF}$. However, for higher SGS severities, $FR_{\text{SGS}}$ increases significantly, causing $FR_{\text{Total}}$ to exceed $FR_{VF}$. Notably, the drop in $FR_{VF}$ for severe cases remains small.

The area ratio results show values below 0.12 for SGS severities of 75% and lower, corresponding to the unchanged flow rate and vocal fold vibration. At 90% SGS severity, the area ratio rises to 0.29, indicating the changed flow rate but minimal changes in vocal fold vibration compared to the baseline. For higher SGS severities and consequently higher area ratios, both the flow rate and vocal fold vibration change noticeably.

These observations suggest that area ratios of 0.12 and lower do not significantly affect $FR_{\text{Total}}$, resulting in the unchanged flow rate and acoustic results. However, for larger area ratios, the change in $FR_{\text{Total}}$ becomes substantial. This implies that in severe SGS cases, both the SGS and vocal fold resistances contribute significantly to altering the flow rate and, consequently, the acoustics. Conceptually, we can consider the airway as a duct with two flow resistances: SGS and vocal fold. Both become significant enough to change the flow rate and acoustics in severe cases. However, the underlying mechanism of the vocal fold vibration changes, which only occurred at the 96% SGS severity case, requires more investigation.

By examining Figure 11a more closely, we observe that the change in ${FR}_{VF}$ for the 96% case is small. Comparing to our previous study, while the drop in ${FR}_{VF}$ for the 96% SGS severity case was slightly larger, the ${FR}_{VF}$ did not change significantly for 96% and 99% SGS severity cases. However, there was a significant drop in the vibration of the 99% case compared to the 96% case. This suggests that ${FR}_{VF}$ alone is not the main parameter affecting vocal fold vibration.

**Figure 12.**
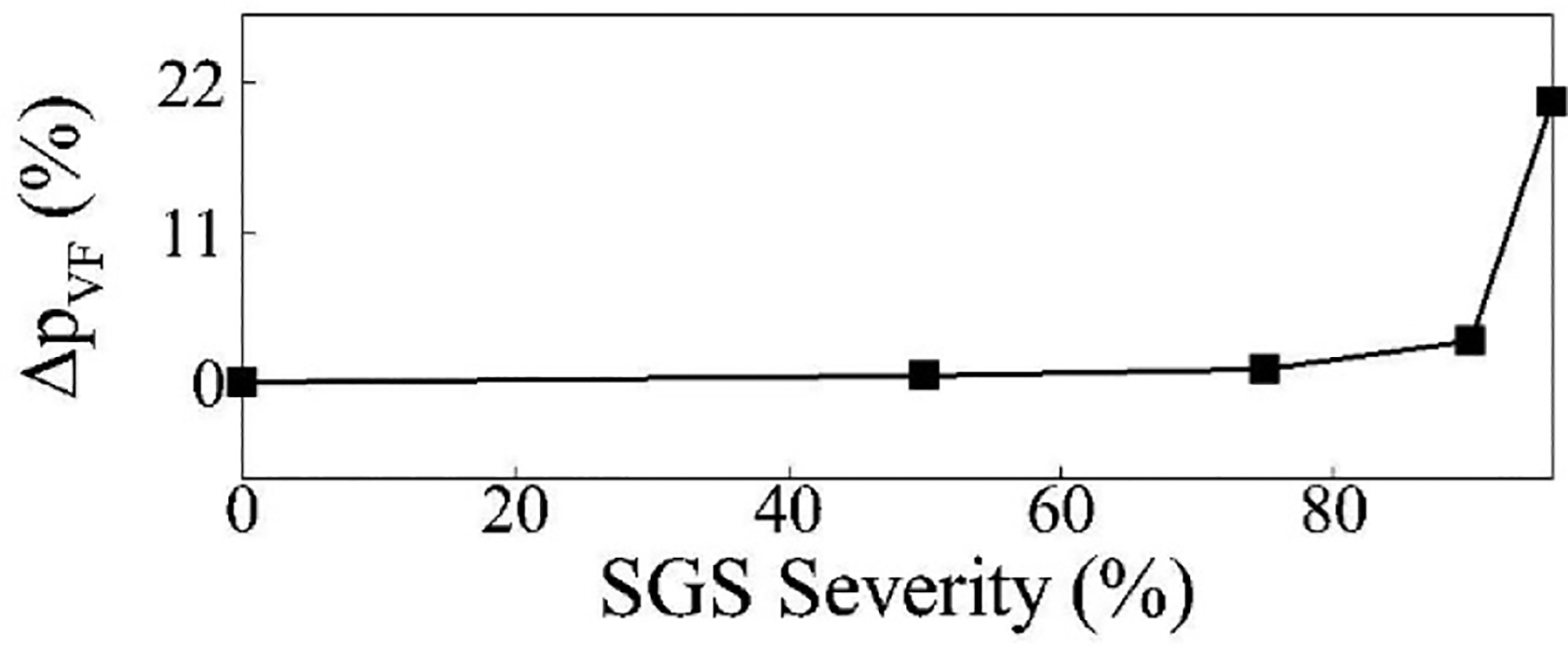
The pressure drop change across the vocal fold compared to the baseline case for each SGS severity case.

To better understand this mechanism, we focus on the pressure drop across the vocal folds $\Delta p_{VF}$) rather than flow resistance, as the pressure of the flow in the glottis is the primary driver of vocal fold vibration. Figure 12 shows the percentage change in pressure drop across the vocal folds for each SGS severity case compared to the baseline, calculated as $\Delta p_{VF}=\frac{\Delta p_{VF,\text{baseline}}-\Delta p_{VF,SGS}}{\Delta p_{VF,\text{baseline}}}\times 100$.

The pressure drop remains nearly unchanged for cases up to 75% SGS severity. For the 90% SGS severity case, $\Delta p_{VF}$ drops by 3%, which, while not causing significant changes in overall vocal fold vibration, does lead to slight changes in the energies of the POD modes as observed earlier. However, for the 96% SGS severity case, the drop in $\Delta p_{VF}$ is much more substantial at 20%.

This analysis helps explain our earlier observations. The slight pressure drop at 90% SGS severity causes minor changes in vibration patterns (reflected in POD mode energy changes) without significantly altering overall vocal fold dynamics. The much larger pressure drop at 96% severity, however, leads to the substantial changes in vocal fold vibration we observed.

Our findings are consistent with previous simplified model studies [14] regarding several fundamental mechanisms, including the threshold of 75% stenosis for initiating significant changes, the progression sequence where flow changes precede vibration changes, the dominant roles of flow resistance and pressure drop, and the relationship between area ratios and onset of changes. Beyond these common features, our patient-specific model revealed distinct differences in severe cases. Particularly, the anterior–posterior opening patterns and focused jet effects led to changes in vocal fold dynamics. As detailed in Section 3.2.2, these changes manifested as a leftward shift in the glottal area waveform (versus rightward in simplified models), decreases in the open quotient (versus increases), and increases in fundamental frequency (versus decreases), along with significant changes in flow acceleration/deceleration duration. These findings provide valuable insights into the progression of SGS effects on voice production, suggesting that while flow changes may occur at lower severities, significant alterations in vocal fold dynamics only manifest at very high stenosis levels.

## 4. Conclusions

This study presents a comprehensive investigation of voice production using a subject-specific model that incorporates realistic geometries and ambient domain. By employing patient-specific geometries, we demonstrate the feasibility of using such simulations for clinical applications, potentially offering more accurate representations of physiological conditions. We employed direct numerical simulation of flow-structure interaction based on compressible Navier–Stokes equations to examine the glottal flow rate, vocal fold dynamics, and acoustics.

Our baseline model effectively captured essential physiological parameters. The characteristics of the glottal flow rate waveform, including the mean and peak flow rates and skewness quotient, along with the glottal area waveform parameters such as fundamental frequency and the open quotient, all fell within established physiological ranges. Additionally, the realistic vocal fold model accurately recreated the convergent-divergent pattern, which is crucial for efficient phonation. Our acoustic analysis also identified the first four formant frequencies of the airway tract with reasonable accuracy when compared to experimental data, further validating our model’s ability to simulate complex vocal tract acoustics.

We extended our study to explore the effects of subglottic stenosis (SGS) on voice production using realistic geometries. Our findings reveal a relationship between SGS severity and various aspects of phonation. Up to 75% SGS severity, we observed no significant changes in glottal flow, vocal fold vibration, or acoustics. At 90% SGS severity, glottal flow was notably impacted, with decreases in the mean and peak flow rates, skewness quotient, and maximum flow declination rate (MFDR). While vocal fold vibration remained largely unchanged, we detected slight alterations in the energies of the Proper Orthogonal Decomposition (POD) modes. The acoustic field exhibited minor decreases in sound pressure level and vocal efficiency. At 96% SGS severity, we noted accelerated declines in glottal flow dynamics and acoustic parameters. Vocal fold dynamics were significantly affected, showing decreases in the mean and peak glottal areas and open quotient, along with an increase in fundamental frequency. POD analysis revealed that the most substantial change in vocal fold vibration occurred in the second mode shape at this severity. It is also noteworthy that while our patient-specific model captures anatomical details and variability with high fidelity, our results suggest that these subtleties do not introduce significant changes in model outputs, especially at lower SGS severities. However, in cases of advanced stenosis (e.g., 90% or 96% obstruction), the effects of patient-specific details in geometry become more evident to accurately capture the progression of aerodynamic and acoustic changes.

Our analysis of the underlying mechanisms revealed that flow resistance—particularly the interaction between resistances at the subglottic stenosis (SGS) and the vocal folds—plays a crucial role in determining the onset and extent of changes in voice production. We found that the ratio of glottal to stenosis area significantly impacts voice production in severe cases. Our study suggested that the pressure drop across the vocal folds, rather than flow resistance alone, is a key factor in altering vocal fold vibration patterns. This was especially evident in the 96% SGS case, where a significant pressure drop led to substantial changes in vocal fold dynamics. Our findings demonstrated that while flow changes may occur at lower severities, significant alterations in vocal fold dynamics manifest only at very high stenosis levels. These insights confirm that the effects of SGS on the glottal flow rate, vocal fold dynamics, and acoustics are negligible for Grade I and II stenosis (up to 70% obstruction), while higher grades produce increasingly significant impacts that may require treatment. These findings are also in good agreement with the recent studies [12–14], regarding the negligible aerodynamic effect of SGS on voice production.

Using patient-specific geometries, we found several aspects that are consistent with previous simplified model studies, including the threshold of 75% stenosis for initiating significant changes, the role of flow resistance and pressure drops, and the overall progression pattern where flow changes precede vibration changes. However, at severe stenosis levels (>90%), the patient-specific model revealed crucial differences, including anterior–posterior opening patterns and focused jet effects. These differences led to changes in the glottal area waveform, open quotient, and fundamental frequency. These findings indicate that while simplified models work well for mild to moderate stenosis (up to 75%), patient-specific details become essential for severe cases.

We would like to note the potential of the hydrodynamics/splitting method for investigating acoustic source fields across SGS severities. Schoder et al. [48] explored acoustic source fields in voice production using a hybrid aeroacoustic method based on a perturbed convective wave equation formulation. Their analysis quantitatively showed that the time derivative of incompressible pressure dominates acoustic generation in voice production. Building on this, investigating changes in acoustic source fields could further improve our understanding of acoustic characteristics of SGS.

## Figures and Tables

**Table 1.** Material properties of the inner layers of the vocal fold tissue. E is the transverse Young's modulus, $E^{\prime }$ is the longitudinal Young's modulus, G is the longitudinal shear modulus, η is the transverse Poisson ratio, $\eta^{\prime }$ is the longitudinal Poisson ratio, and VSG is the vertical stiffness gradient.

Inner Layer	E (kPa)	$E^{'}$ (kPa)	G (kPa)	μ	$\mu^{'}$	VSG (kPa/mm)
Cover	1.33	26.70	6.68	0.9	0.0	0.43
Body	4.76	95.24	23.81	0.9	0.0	-

**Table 2.** Normal human phonation quantities computed from the realistic geometry simulation and their typical physiological ranges [38]. $f_{0}$ is the fundamental frequency, $\tau_{s}$ is the skewness quotient, $\tau_{0}$ is the open quotient, $q_{\text{peak}}$ is the peak glottal flow rate, $q_{\text{mean}}$ is the mean glottal flow rate and MFDR is the maximum flow declination rate.

	Computed Value	Typical Range [38]
$f_{0}(\text{Hz})$	154	65–260
$\tau_{s}$	1.32	1.1–3.4
$\tau_{0}$	0.69	0.4–0.7
$q_{\text{peak}}\left(\frac{\text{mL}}{\text{s}}\right)$	388	200–580
$q_{\text{mean}}\left(\frac{\text{mL}}{\text{s}}\right)$	198	110–220
$\text{MFDR}\left(\frac{\text{L}}{{\text{s}}^{2}}\right)$	481	-

## Data Availability

The data that support the findings of this study are available from the corresponding author upon reasonable request. The data are not publicly available due to data volume and complexity.
